# Anesthetic management of a patient with an electroencephalogram phenotype for a “vulnerable brain”: a case report

**DOI:** 10.1186/s40981-023-00616-w

**Published:** 2023-05-17

**Authors:** Ryo Wakabayashi

**Affiliations:** Department of Anesthesia, Nagano Red Cross Hospital, 5-22-1, Wakasato, Nagano, 380-8582 Japan

**Keywords:** Electroencephalogram, Minimum alveolar concentration, Postoperative delirium, Vulnerable brain

## Abstract

**Background:**

Low frontal alpha power is an electroencephalogram phenotype suggesting vulnerability to anesthetics. This phenotype for a “vulnerable brain” carries risks for burst suppression at lower-than-expected anesthetic concentrations and therefore for postoperative delirium.

**Case presentation:**

A 73-year-old man underwent a laparoscopic Miles’ operation. He was monitored with a bispectral index monitor. Before the skin incision, the fraction of age-adjusted minimum alveolar concentration of desflurane was 0.48, and a spectrogram showed slow-delta oscillation despite a bispectral index value of 38–48. Although the fraction of age-adjusted minimum alveolar concentration of desflurane decreased to 0.33, the EEG signature remained unchanged, along with a similar bispectral index value. No burst suppression patterns were observed throughout the whole procedure, and he did not experience postoperative delirium.

**Conclusions:**

This case suggests that monitoring of electroencephalogram signatures is helpful for detecting patients with a “vulnerable brain” and for providing optimal anesthetic depth in such patients.

## Background

Postoperative delirium (POD) is associated with longer hospital stay, increased need for long-term care, loss of functional independence, reduced cognition, and death [1]; thus, prevention of POD is very important. Burst suppression on an electroencephalogram (EEG) is a state of profound brain inactivation that can be caused by an excessive dosage of anesthetics, and an association between intraoperative burst suppression and POD has been reported [2]. Low frontal alpha wave power under general anesthesia is an atypical EEG phenotype called “vulnerable brain” [3]. Such patients have the propensity for intraoperative burst suppression at lower-than-expected anesthetic concentrations and thus potentially have an increased risk for POD [3]. Here, I report a patient with “vulnerable brain” diagnosed by EEG signatures on a bispectral index (BIS) monitor (A-3000; Medtronic, Minneapolis, MN) who received low-dose desflurane anesthesia and had neither intraoperative burst suppression nor POD. Written informed consent for publication of this case report was obtained from the patient.

## Case presentation

A 73-year-old male patient (height, 150 cm; weight, 41 kg) underwent a laparoscopic Miles’ operation for rectal cancer. He suffered from heart failure after myocardial infarction and had insulin-treated type 2 diabetes mellitus. Preoperative laboratory investigations indicated anemia with a hemoglobin level of 10.8 g/dl and increased ventricular load with an elevated serum brain natriuretic peptide level of 325.0 pg/ml. A chest X-ray showed clear lung fields with a cardiothoracic ratio of 0.42. An electrocardiogram showed sinus rhythm at 64 bpm with complete right bundle branch block and ST-segment elevations of 1–2 mm in the anterior leads. Transthoracic echocardiography showed impaired left ventricular function with an ejection fraction of 33% and severe hypokinesis of the antero-septal wall. Neurocognitive disorder was not evident in the preoperative period.

**Fig. 1:**
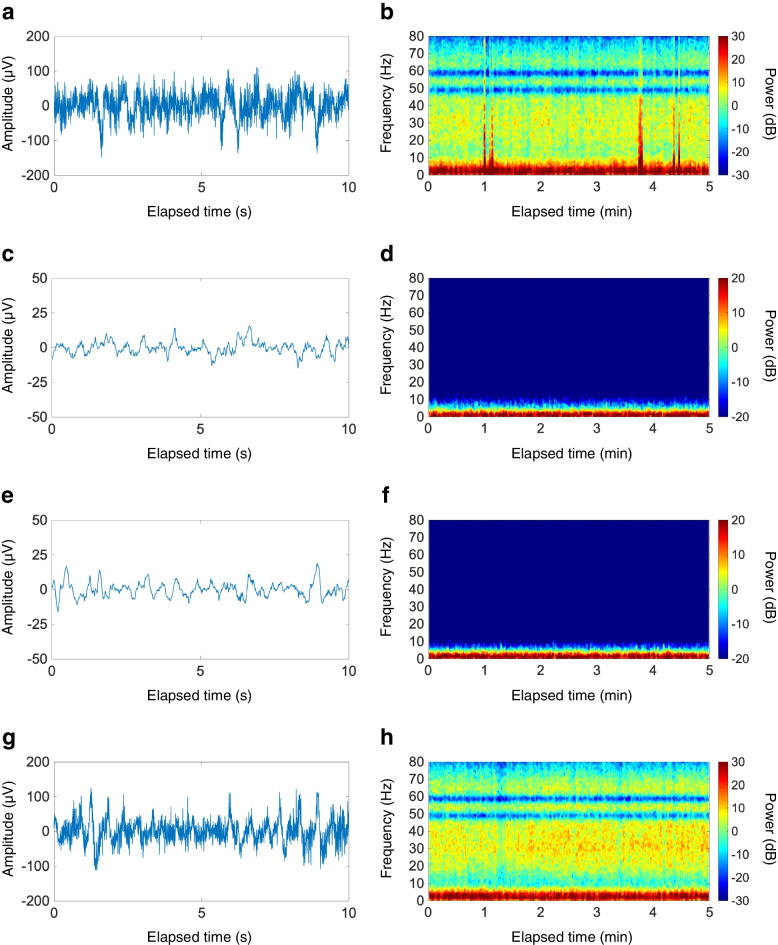
Frontal electroencephalograms (EEGs) and spectrograms obtained by a bispectral index (BIS) monitor. An EEG and a spectrogram before induction of anesthesia, showing no particularity, with a BIS value of 97, spectral edge frequency 95 (SEF95) of 27.4 Hz, electromyogram (EMG) of 67.2 dB, suppression ratio (SR) of 0%, slow-delta power of 51.0 dB, theta power of 39.5 dB, alpha power of 36.9 dB, beta power of 22.0 dB, and gamma power of 12.5 dB (**a**, **b**). An EEG and a spectrogram at the fraction of age-adjusted minimum alveolar concentration (MAC) of desflurane of 0.48 [4] with predicted effect site concentrations of remifentanil and fentanyl of 6.0–6.1 ng/ml [5] and 0.5–1.1 ng/ml [6], respectively, showing low amplitude waveforms and slow-delta oscillation, with a BIS value of 40, SEF95 of 7.6 Hz, EMG of 25.7 dB, SR of 0%, slow-delta power of 12.7 dB, theta power of − 13.2 dB, alpha power of − 20.1 dB, beta power of − 41.8 dB, and gamma power of − 60.3 dB (**c**, **d**). An EEG and a spectrogram at the fraction of age-adjusted MAC of desflurane of 0.33 [4] with predicted effect site concentrations of remifentanil and fentanyl of 6.1–6.2 ng/ml [5] and 0.3–1.5 ng/ml [6], respectively, showing low amplitude waveforms and slow-delta oscillation, with a BIS value of 46, SEF95 of 5.2 Hz, EMG of 25.0 dB, SR of 0%, slow-delta power of 13.7 dB, theta power of − 13.3 dB, alpha power of − 22.2 dB, beta power of − 45.7 dB, and gamma power of − 61.0 dB (**e**, **f**). An EEG and a spectrogram after extubation with predicted effect site concentrations of remifentanil and fentanyl of 0.3–0.4 ng/ml [5] and 1.0–1.1 ng/ml [6], respectively, showing no particularity, with a BIS value of 91, SEF95 of 24.5 Hz, EMG of 64.9 dB, SR of 0%, slow-delta power of 39.3 dB, theta power of 20.8 dB, alpha power of 11.6 dB, beta power of 15.0 dB, and gamma power of 9.2 dB (**g**, **h**)

On the day of surgery, the patient received no premedication. A BIS Quatro sensor (Medtronic, Minneapolis, MN) was placed on the forehead and was connected to a BIS A-3000 monitor, and the electrode impedance was maintained at 5 kΩ or lower throughout the whole procedure. EEG data were collected by a PrimeGaia system (Nihonkohden, Tokyo, Japan) at a sampling rate of 250 Hz and analyzed with MATLAB R2020a (Math- works, Natick, MA). A power spectrum and spectrogram were obtained by using the Chronux *mtspecgramc* function and using the following parameters: window length, 2 s with 0.1-s overlapping; time-bandwidth product, 2; and number of tapers, 3. Before induction of anesthesia, an EEG on the BIS monitor showed no particularity (Fig. 1A, B). General anesthesia was induced with 1 mg/kg propofol and 0.2 μg/kg/min remifentanil. After 1.2 mg/kg rocuronium had been intravenously administered, the trachea was intubated, and the patient’s lungs were mechanically ventilated. Anesthesia was maintained with 2–3% desflurane, 0.25 μg/kg/min remifentanil, and intermittent bolus of fentanyl (total of 10 μg/kg). After induction of anesthesia, transversus abdominis plane block and rectus sheath block were performed using 1.2 ml/kg 0.25% ropivacaine. During anesthesia, nasopharyngeal temperature ranged from 36.0 to 37.1℃ and mean blood pressure was maintained at 68–88 mmHg with 0.25 μg/kg/min phenylephrine.

Before the skin incision, the end-tidal concentration of desflurane was 2.6% (fraction of age-adjusted minimum alveolar concentration [MAC] of 0.48 [4]) and the predicted effect site concentrations of remifentanil and fentanyl were 6.0–6.1 ng/ml [5] and 0.5–1.1 ng/ml [6], respectively. At that time, an EEG on the BIS monitor and its spectrogram showed slow-delta oscillation with very low theta, alpha, beta, and gamma power (Fig. 1C, D) and the BIS value ranged from 38 to 48. The end-tidal concentration of desflurane was gradually tapered to 1.8% (fraction of age-adjusted MAC of 0.33 [4]) under the predicted effect site concentrations of remifentanil and fentanyl of 6.1–6.2 ng/ml [5] and 0.3–1.5 ng/ml [6], respectively. However, the EEG signature remained unchanged (Fig. 1E, F), along with similar BIS values of 35–50. No burst suppression patterns were observed throughout the whole procedure.

The surgery was completed uneventfully in 392 min. The total amount of fluid infusion was 1700 ml, total blood loss was 20 ml, and total urine volume was 600 ml. The patient recovered from anesthesia 9 min after termination of desflurane administration. After uneventful extubation, an EEG on the BIS monitor showed no particularity (Fig. 1G, H) under the predicted effect site concentrations of remifentanil and fentanyl of 0.3–0.4 ng/ml [5] and 1.0–1.1 ng/ml [6], respectively. He did not experience intraoperative awareness with explicit recall. Postoperative analgesia was effectively provided by intravenous infusion of 0.5 μg/kg/h fentanyl. The patient had an uneventful postoperative course without complications including POD and was discharged on postoperative day 27.

## Discussion

The prominent signature of a frontal EEG under surgical level anesthesia with gamma-aminobutyric acid (GABA) type A receptor-mediated anesthetics including desflurane is dominant slow-delta and alpha oscillations [7]. GABAergic anesthetics-induced frontal alpha oscillation is thought to be generated by thalamocortical loop mechanisms [7], and the putative prefrontal cortical generators for frontal alpha oscillation might be susceptible to thinning of the cortex [8]. Shao et al. showed that there is significant variation in GABAergic anesthetics-induced frontal alpha power across individuals at a given age and that a low frontal alpha power is an EEG phenotype for a “vulnerable brain” above and beyond a patient’s chronological age [3]. In the current case, frontal alpha power was very low during sub-MAC desflurane anesthesia. Frontal alpha power is also attenuated in specific conditions including hypothermia [7] and cerebral ischemia [9]. In the present case, nasopharyngeal temperature and mean arterial blood pressure were maintained throughout the whole procedure. Although administration of remifentanil and fentanyl leads to EEG slowing in a dose-dependent manner [10, 11], the doses of remifentanil and fentanyl in this patient might not have conferred significant effects on frontal alpha power [12]. Therefore, it is conceivable that the very low frontal alpha power observed in the patient was derived from a “vulnerable brain.”

Currently, monitoring during general anesthesia maintained with a volatile anesthetic includes real-time measurement of its fraction of age-adjusted MAC [13]. With any volatile anesthetic, keeping the fraction of age-adjusted MAC greater than or equal to 0.7 helps to minimize the risk of intraoperative awareness with explicit recall while at the same time reducing the incidence of severe overdosing [13]. However, this patient showed an EEG signature of slow-delta oscillation with very small theta, alpha, beta, and gamma waves under the condition of a fraction of age-adjusted MAC of 0.48. This EEG signature suggested not only that there is a “vulnerable brain” [3] but also that desflurane is not underdosing [7]. Therefore, a further increase of the desflurane dosage was deemed unnecessary and low-dose desflurane anesthesia was provided.

When the fraction of age-adjusted MAC has to be decreased to less than 0.7, determining the depth of anesthesia by EEG-derived indices may be helpful [13]. A BIS monitor is an established device for measuring anesthetic depth, and the BIS value is now the most common parameter for monitoring depth of anesthesia [14]. However, the BIS value relies on power and relative power in the slow-delta and alpha oscillations [15]. As mentioned above, desflurane shows slow-delta and alpha oscillations at sub-MAC concentrations [7]. However, this patient showed very low frontal alpha power under the condition of a fraction of age-adjusted MAC of 0.33–0.48. Thus, the BIS values observed in this patient might have been unreliable because of this atypical EEG phenotype with very low alpha waves.

Intraoperative awareness with explicit recall is a serious complication of anesthetic practice that is associated with a high rate of posttraumatic stress disorder [16]; therefore, its prevention is of critical importance. The threshold of the fraction of age-adjusted MAC that would ensure lack of awareness with explicit recall probably lies above 0.3 [16]. In this patient, desflurane was thus tapered up to a fraction of age-adjusted MAC of 0.33. Nevertheless, the EEG signature was slow-delta oscillation with very small theta, alpha, beta, and gamma waves even under such low-dose desflurane anesthesia. On emergence from desflurane anesthesia, there is dissipation of slow-delta and alpha oscillation power, followed by reappearance of the power in the beta and gamma bands [7]. Therefore, anesthetic depth was possibly sufficient despite the fraction of age-adjusted MAC of 0.33, resulting in the absence of intraoperative awareness with explicit recall.

In the present case, desflurane was titrated on the basis of the EEG signature. As a consequence, the patient did not have intraoperative burst suppression, POD, and intraoperative awareness with explicit recall. A recently proposed anesthetic approach is interpretation of an unprocessed EEG and spectrogram [7]. In this patient, the spectrogram readily visualized the EEG signature and contributed to patient care by showing not only the underlying brain state but also the instantaneous depth of anesthesia. Further accumulation of cases with “vulnerable brain” is required to improve anesthetic management of such patients.

In conclusion, the present case suggests that intraoperative monitoring of EEG signatures is helpful for detecting patients with “vulnerable brain” and for providing optimal anesthetic depth in such patients.

## Data Availability

Not applicable.

## Abbreviations

BIS: Bispectral index
EEG: Electroencepharogram
GABA: Gamma-aminobutyric acid
MAC: Minimum alveolar concentration
POD: Postoperative delirium
